# Genome-wide identification of WOX transcription factors and functional characterization of WOX4 and WOX13 involved in cold stress response *in Malus baccata*

**DOI:** 10.3389/fgene.2026.1920502

**Published:** 2026-08-28

**Authors:** Yanting Tian, Hao Liu, Fei Wang, Lifang Yang, Xuhong Zhang, Huanyong Li, Xin Qi, Jing Guo

**Affiliations:** 1 The Research Institute of Forestry and Pomology, Tianjin Academy of Agricultural Sciences, Tianjin, China; 2 Shijiazhuang Landscape Management and Maintenance Center, Shijiazhuang, China; 3 Institute of Economic Forestry, Xinjiang Uygur Autonomous Region Academy of Forestry Sciences, Urumqi, China

**Keywords:** cold stress, gene function, genome-wide identification, *Malus baccata*, WOX transcription factor

## Abstract

**Introduction:**

Cold stress is a major abiotic threat to apple production. *Malus baccata* has exceptional cold hardiness and is widely used as a superior cold-resistant rootstock. The WUSCHEL-related homeobox (WOX) transcription factor family regulates plant growth, development and stress adaptation, whereas the functions of WOX genes in cold tolerance of *M. baccata* remain elusive.

**Methods:**

In the present work, 19 MbWOX family members were identified and characterized at the genome-wide level. Evolutionary analysis, cis-element prediction, transcriptome profiling and real-time quantitative PCR (RT-qPCR) were performed to screen core cold-responsive genes. Overexpression vectors were constructed and transformed into Arabidopsis seedlings for functional verification.

**Results:**

Evolutionary analysis revealed that segmental duplication drove the expansion of the MbWOX family, and these genes contained a variety of stress-responsive cis-elements. Combined transcriptome and RT-qPCR analyses confirmed that *MbWOX4* and *MbWOX13* were core cold-responsive genes with distinct expression patterns. The two genes participated in cold signal transduction by interacting with different transcription factor networks. Functional tests revealed that *MbWOX4* and *MbWOX13* isoforms differentially modulated seedling cold tolerance under low-temperature stress.

## 1 Introduction

The WUSCHEL-related homeobox (WOX) gene family consists of plant-specific transcription factors characterized by a conserved homeodomain (HD). They exert pivotal regulatory effects on multiple vital processes of plant growth and development. The first WOX gene, WUSCHEL (WUS), was identified in *Arabidopsis thaliana* in 1996. It participates in the maintenance of shoot apical meristem (SAM), and its loss of function leads to premature termination of meristem differentiation (Laux et al., 1996). Since then, WOX genes have been widely identified and investigated in numerous plant species, including Arabidopsis, rice, maize, soybean and poplar. Distinct differences exist in member number, phylogenetic relationship and biological functions of WOX genes across various species, indicating that this gene family has undergone expansion and differentiation during plant evolution to adapt to species-specific growth demands and environmental adaptability. The Arabidopsis genome harbors 15 AtWOX members, classified into ancient, intermediate and modern clades based on phylogenetic analysis (Haecker et al., 2004). 13 OsWOX genes, 14 ZmWOX genes and 33 GmWOX genes have been characterized in rice, maize and soybean respectively, reflecting the expansion tendency of WOX family in dicotyledons (van der Graaff et al., 2009; Cheng et al., 2014; Hao et al., 2019). The N-terminal HD domain composed of approximately 60 amino acid residues serves as the core conserved structure of WOX proteins, which can specifically bind to cis-elements in the promoter regions of downstream target genes and modulate gene transcription (Mayer et al., 1998). Several WOX proteins also contain C-terminal conserved motifs such as WUS-box and EAR motif. WUS-box is responsible for meristem maintenance, while EAR motif functions in transcriptional repression (Kieffer et al., 2006). WOX genes are predominantly expressed in meristems (shoot apical meristem, root apical meristem and lateral meristem) and embryos (Dong et al., 2024). They are extensively involved in embryonic development, meristem homeostasis, organogenesis, flower and fruit formation, as well as apical dominance regulation (Haecker et al., 2004; Du et al., 2025). For instance, *AtWUS* forms a negative feedback loop with *CLV3* to sustain SAM stability (Zhou et al., 2018; Dong et al., 2026). In Arabidopsis, *AtWOX2* is regulated by *AtWOX8* and *AtWOX9*, and is required to form the SAM during embryonic development (Breuninger et al., 2008). *AtWOX1*, *AtWOX3*, *AtWOX5*, *AtWOX8*, and *AtWOX9* are redundantly expressed together with *AtWOX2* during SAM formation (Breuninger et al., 2008). *AtWOX5* is specifically expressed in the quiescent center of root apical meristem and regulates root growth (Sarkar et al., 2007). Rice *OsWOX4* modulates vascular tissue differentiation and root elongation (Ohmori et al., 2013; Yasui et al., 2018; Yang et al., 2023). In Arabidopsis, the genes of the ANCIENT clade *WOX13* and *WOX14* are expressed in primary roots, lateral roots, and floral organs, precluding premature cell differentiation (Deveaux et al., 2008).

Beyond regulating fundamental plant growth and development, WOX genes also extensively respond to biotic and abiotic stresses. In rice, overexpression of the *OsWOX13* gene under the control of the *rab21* promoter increases the tolerance to drought (Minh-Thu et al., 2018). In *Gossypium hirsutum*, over half of WOX genes show no significant response to heat, cold, salinity, or drought. By contrast, the *GhWOX10_Dt*, *GhWOX13a_At/Dt*, and *GhWOX13b_At/Dt* genes are strongly induced under multiple stress (Yang et al., 2017). In *Brassica napus*, 58 WOX genes have been identified and characterized. Analysis of their expression levels showed that four members belonging to the WOX4 sub-clade (*BnWOX10*, *BnWOX50*, *BnWOX44*, and *BnWOX1*8) are activated by abiotic stress or PGR treatments (Han et al., 2021). In Arabidopsis, *AtWOX6* prevents premature differentiation during the formation of the integuments that envelop the embryonic sac and the egg cell (Park et al., 2005). A further role of *AtWOX6* has been identified through the isolation of the *hos9-1* mutant, characterized by slower growth and late flowering, with an accentuated sensitivity to low temperatures that leads the plant to undergo extensive freezing phenomena (van der Graaff et al., 2009; Peng et al., 2015; Fambrini et al., 2022). In wheat, *TaWOX5* improves the activities of antioxidant enzymes including superoxide dismutase (SOD) and peroxidase (POD), eliminates excess ROS and reduces malondialdehyde (MDA) content (Wang et al., 2022; Chang et al., 2024). It also upregulates cold-related genes such as *TaCOR14* and *TaRD29A*, thereby strengthening plant cold resistance (Chang et al., 2024).

Cold stress is one of the major abiotic stresses that restricts plant growth, geographical distribution and crop yield. Low temperature impairs cell membrane integrity, induces reactive oxygen species (ROS) accumulation, and inhibits photosynthesis and metabolic activities, which may cause plant death under severe conditions (Sharma et al., 2012; Pospíšil, 2016; Yang et al., 2023). Plants have developed complex mechanisms to defend against cold stress, in which transcription factors play indispensable roles. Numerous molecular studies have demonstrated that cold stress markedly induces the expression of WOX family genes, whose members mediate plant cold tolerance through diverse regulatory pathways. WOX genes regulate the expression of cold-responsive genes, modulate hormone signaling pathways, sustain cellular homeostasis and preserve meristem activity. Yet the functions of distinct WOX members in cold tolerance are poorly understood.

*Malus baccata*, a native wild apple relative endemic to northern China, possesses extraordinary cold resistance and can survive temperatures ranging from −25 °C to −35 °C. It is widely used as a dominant cold-resistant rootstock for apple cultivation, with favorable grafting compatibility and high propagation efficiency, and can effectively improve cold adaptability of apple tree (Yang et al., 2008; Kashyap and Forestry, 2014; Li et al., 2019). Currently, the expression patterns and molecular regulatory mechanisms of WOX genes in *M. baccata* cold response are still insufficiently studied, and key functional WOX members have not been clarified, limiting their application in cold-resistant apple rootstock molecular breeding. In this study, *M. baccata* was used as experimental material. WOX family members were systematically identified, and their phylogenetic relationships, gene structures, conserved motifs and expression profiles under different cold temperatures and treatment durations were analyzed. *MbWOX4* and *MbWOX13* were screened as core candidate genes participating in cold regulation. Overexpression vectors of the two genes were constructed and transformed into Arabidopsis seedlings for functional verification. Both *MbWOX4* and *MbWOX13* were significantly upregulated under cold stress, and their expression levels increased first and then stabilized, reaching the peak under extreme low temperature of −40 °C. Transgenic seedlings overexpressing *MbWOX4b*, *MbWOX13a* and *MbWOX13b* exhibited distinctly enhanced cold resistance. The findings reveal the core mechanism of WOX genes regulating cold response in *M. baccata*, and confirm the critical roles of *MbWOX4* and *MbWOX13*. This study provides novel molecular targets and theoretical strategies for cold resistance improvement of Malus species, excavation and utilization of elite cold-tolerant gene resources, and molecular breeding of cold-resistant apple rootstocks. It also deepens the understanding of strong cold adaptation mechanism of *M. baccata*, and contributes to high-quality development of cold-region apple industry and ecological restoration.

## 2 Methods

### 2.1 Genome-wide identification of the WOX gene family

The Hidden Markov Model (HMM) corresponding to the conserved HD domain of WOX proteins was downloaded from the Pfam database (http://pfam.xfam.org). The whole-genome protein sequences of *M. baccata* (accession: GCA_054553115.1_ASM5455311v1) were screened with HMMER 3.0 software to collect candidate WOX homologs. All candidate sequences were further validated against the NCBI Conserved Domain Database (CDD). Sequences that did not carry an intact HD domain were discarded, and the remaining sequences were defined as the final members of the MbWOX gene family.

### 2.2 Physicochemical properties and chromosomal localization of MbWOX proteins

TBtools software was applied to extract the full-length protein sequences of all MbWOX members. The ProtParam online tool was adopted to characterize their basic physicochemical features, including amino acid number, molecular weight, theoretical isoelectric point, protein instability index and grand average of hydropathicity (GRAVY). The genomic GFF3 annotation file was loaded into TBtools to map the distribution of MbWOX genes on chromosomes.

### 2.3 Multiple sequence alignment and phylogenetic analysis

WOX protein sequences of *A. thaliana*, *Populus trichocarpa*, *Prunus persica* and *Malus domestica* were retrieved from public biological databases. Multiple sequence alignment was carried out via the ClustalW program embedded in MEGA11. A phylogenetic tree was then built with the Neighbor-Joining (NJ) algorithm. During tree construction, the Poisson substitution model and pairwise deletion mode were selected to divide MbWOX genes into different evolutionary clades.

### 2.4 Conserved motif, gene structure, and domain analysis

The MEME web server (https://meme-suite.org/meme) was used to predict conserved motifs across MbWOX proteins, while the NCBI Batch CD-Search platform (https://www.ncbi.nlm.nih.gov/Structure/bwrpsb/bwrpsb.cgi) was employed for conserved domain annotation. Relevant gene structure data were acquired from the genomic GFF3 file. TBtools was finally used to realize the integrated visualization of conserved motifs, functional domains and exon-intron structures of MbWOX genes.

### 2.5 Cis-acting regulatory element analysis and collinearity analysis

For each MbWOX gene, the 2000 bp sequence upstream of the transcription start site was isolated and uploaded to the PlantCARE database (http://bioinformatics.psb.ugent.be/webtools/plantcare/html/). We mainly screened cis-acting elements associated with low temperature, phytohormone and light signaling. TBtools was used to conduct intra-genomic collinearity analysis of *M. baccata*, as well as interspecific collinearity comparison between *M. baccata* and two Rosaceae species (*Malus domestica* and *Prunus persica*). The Ka/Ks values of homologous gene pairs were calculated to assess natural selection pressure.

### 2.6 Construction of overexpression vectors and genetic transformation

The CDS sequences of *MbWOX4a/b* and *MbWOX13a/b* were amplified by PCR and cloned into overexpression vectors (PBI121). The recombinant plasmids were transformed into *Agrobacterium tumefaciens* strain GV3101. Col-0 Arabidopsis transformation was performed using the floral dip method with GV3101. Positive overexpression lines were identified by genomic PCR and RT-qPCR. Progeny screening was performed on kanamycin-containing medium followed by PCR verification. After two rounds of screening, F2 generation transgenic lines were obtained.

### 2.7 Total RNA extraction and RT-qPCR analysis

Total RNA was extracted from each sample using a plant total RNA extraction kit. First-strand cDNA was synthesized using the HiScript III Reverse Transcription Kit. RT-qPCR was performed using the SYBR Green method to analyze the relative expression levels of MbWOX genes under low-temperature stress, with a housekeeping gene used as the internal reference for normalization. MbActin-F-ACACGGGGAGGTAGTGACAA, MbActin-R-CCTCCAATGGATCCTCGTTA.

### 2.8 Time*-*ordered gene co-expression network (TO-GCN) analysis

The construction of TO-GCNs was conducted with gene expression matrices from four groups (0 h, 12 h, 24 h, 36 h, 48 h, 72 h and 10 days) as input (Li et al., 2024; Dai et al., 2025). Pearson’s correlation coefficient (PCC) values were calculated for each pair of genes. The PCC cutoff value of 0.83 was applied for interactions between TF and non-TF genes. With this threshold, a TO-GCN was constructed in the ‘C1 + C2 +’ mode, following established procedure. To generate hierarchical levels of TO-GCN, gene-C1H46_021534 with consistent expression trends from 0 h to 10 days were selected as seed genes for subsequent analysis.

To construct the hierarchical Time-Ordered Gene Coexpression Network (TO-GCN), seed genes with continuously declining expression from 0 h to 10 days were required. We adopted MFSelector software developed by the research team that established the TO-GCN method, and finally screened out 20 optimal candidate seed genes. Among them, gene C1H46_021534 is annotated as *NF-YA3* (nuclear factor Y, subunit A3), which shows the highest expression level at the 0 h time point and meets the criteria for TO-GCN construction.

### 2.9 Phenotypic observation and low-temperature stress treatment

Wild-type and overexpression seedlings with consistent growth status were subjected to low-temperature treatment at 0 °C. The degree of vitrification, growth status, survival rate, and other phenotypes were observed and recorded, and photographs were taken.

## 3 Results

### 3.1 Identification and physicochemical characterization of MbWOX gene family members in *M. baccata*

Based on the conserved domains of WOX proteins, HD, a total of 19 WOX genes were identified from M. baccata. According to sequence homology with the 15 previously identified WOXgenes in *A. thaliana*, the *WOX*genes from *M. baccata* were named as MbWOX. The gene ID of the MbWOX genes are listed in Table 1. The predicted lengths of the MbWOX proteins range from 176 amino acids (*MbWOX5a*) to 843 amino acids (*MbWOX17*) (Table 1). Their molecular weights were estimated to vary from 25,548.3 Da (*MbWOX11a*) to 92,801.27 Da (*MbWOX17*). The theoretical isoelectric points (pI) range from 4.85 (*MbWOX13a*) to 9.26 (*MbWOX4a*), suggesting a wide diversity in protein charge properties. All proteins exhibited instability index (II) values greater than 40, ranging from 48.5 (*MbWOX15*) to 78.64 (*MbWOX3*), indicating that these proteins are likely unstable *in vitro*. The aliphatic index (AI) values ranged from 49.39 (*MbWUS*), implying lower thermostability, to 89.89 (MbWOX15), indicating higher thermostability. The grand average of hydropathicity (GRAVY) values ranged from −0.947 (*MbWOX13c*) to −0.081 (*MbWOX15*). All values were negative, suggesting that the MbWOX proteins are hydrophilic and likely soluble in water.

**TABLE 1:** The information of the number of amino acid, molecular weight (MWs), theoretical pI (pI), Instability Index (II), Aliphatic Index (AI) and Grand Average of Hydropathicity (GRAVY) of MbWOX.

Gene ID	Name	Number of amino acid	MWs (Da)	pI	II	AI	GRAVY
MABA029828	*MbWOX5a*	176	20,181.74	8.76	58.45	68.12	−0.702
MABA016159	*MbWOX13a*	216	24,876.67	4.85	62.18	61.85	−0.918
MABA002492	*MbWOX2*	230	25,933.26	9.04	73.17	63.17	−0.693
MABA020334	*MbWOX11a*	230	25,548.3	6.79	77.01	62.3	−0.68
MABA015243	*MbWOX3*	237	27,190.59	8.75	78.64	57.26	−0.799
MABA027545	*MbWOX4a*	239	26,704.38	9.26	54.82	66.49	−0.72
MABA040859	*MbWOX13b*	267	30,706.23	5.64	64.2	58.84	−0.931
MABA003596	*MbWOX4b*	272	30,370.9	6.67	54.65	60.22	−0.865
MABA008221	*MbWOX13c*	276	31,548.06	5.62	60.55	57.25	−0.947
MABA010069	*MbWOX11b*	284	30,861.43	5.76	59.14	80.6	−0.297
MABA033613	*MbWUS*	328	36,146.43	6.67	59.31	49.39	−0.827
MABA004520	*MbWOX1b*	380	43,317.21	7.74	56.31	57.08	−0.811
MABA023754	*MbWOX1a*	381	43,074.99	7.28	56.56	57.48	−0.773
MABA026336	*MbWOX9b*	400	44,107.03	7.44	59.05	64.6	−0.586
MABA013378	*MbWOX9a*	409	45,226.26	7.19	57.56	64.87	−0.588
MABA041301	*MbWOX5b*	424	47,968.52	6	49.42	88.47	−0.451
MABA035476	*MbWOX16*	824	90,466.69	5.84	51.17	84.55	−0.136
MABA018994	*MbWOX15*	838	92,067.88	6.14	48.5	89.89	−0.081
MABA019548	*MbWOX17*	843	92,801.27	5.9	51.12	84.15	−0.161

### 3.2 MbWOX genes phylogenetic analysis

**FIGURE 1:**
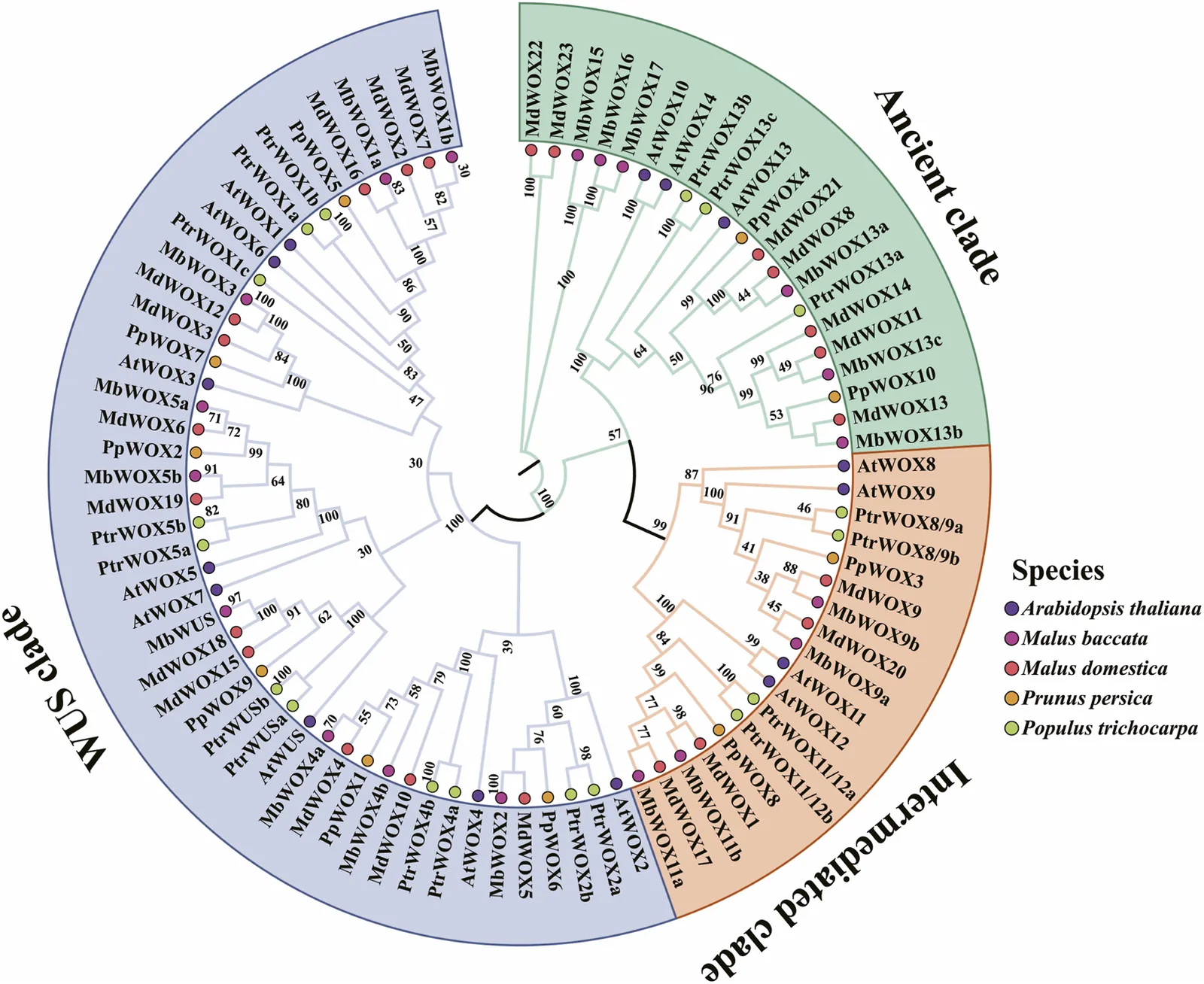
Neighbor-Joining phylogenetic tree of WOX full-length protein sequences from five plant species. A total of 93 protein sequences were used for tree construction, including 19 MbWOX protein, as well as 15 AtWOX, 14 PtrWOX, 10 PpWOX and 24 MdWOX proteins. The WOX family was divided into three evolutionary clades. Species abbreviations: At = *Arabidopsis thaliana*, Ptr = *Populus trichocarpa*, Mb = *Malus baccata*, Md = *Malus domestica*, Pp = *Prunus persica*.

To explore the evolutionary relationships of WOX proteins in *M. baccata* with those from other plant species, a Neighbor-Joining (NJ) phylogenetic tree was built based on a total of 93 full-length protein sequences (Figure 1). In this tree, we included 19 MbWOX protein identified in the *M. baccata* genome; the remaining protein sequences were retrieved from *A. thaliana* (15), *P. trichocarpa* (14), *P. persica* (10) and *M. domestica* (24). The unrooted tree divided the WOX family into three major clades: the WUS Clade, Intermediate Clade and Ancient Clade. The 19 MbWOX protein isoforms were distributed as 9 variants in the WUS Clade, 4 in the Intermediate Clade and 6 in the Ancient Clade. The WUS Clade harbored the greatest number of MbWOX protein isoforms (9), implying lineage-specific expansion and potential functional divergence of MbWOX paralogs during the evolution of *M. baccata*.

### 3.3 Chromosomal location of MbWOX genes

**FIGURE 2:**
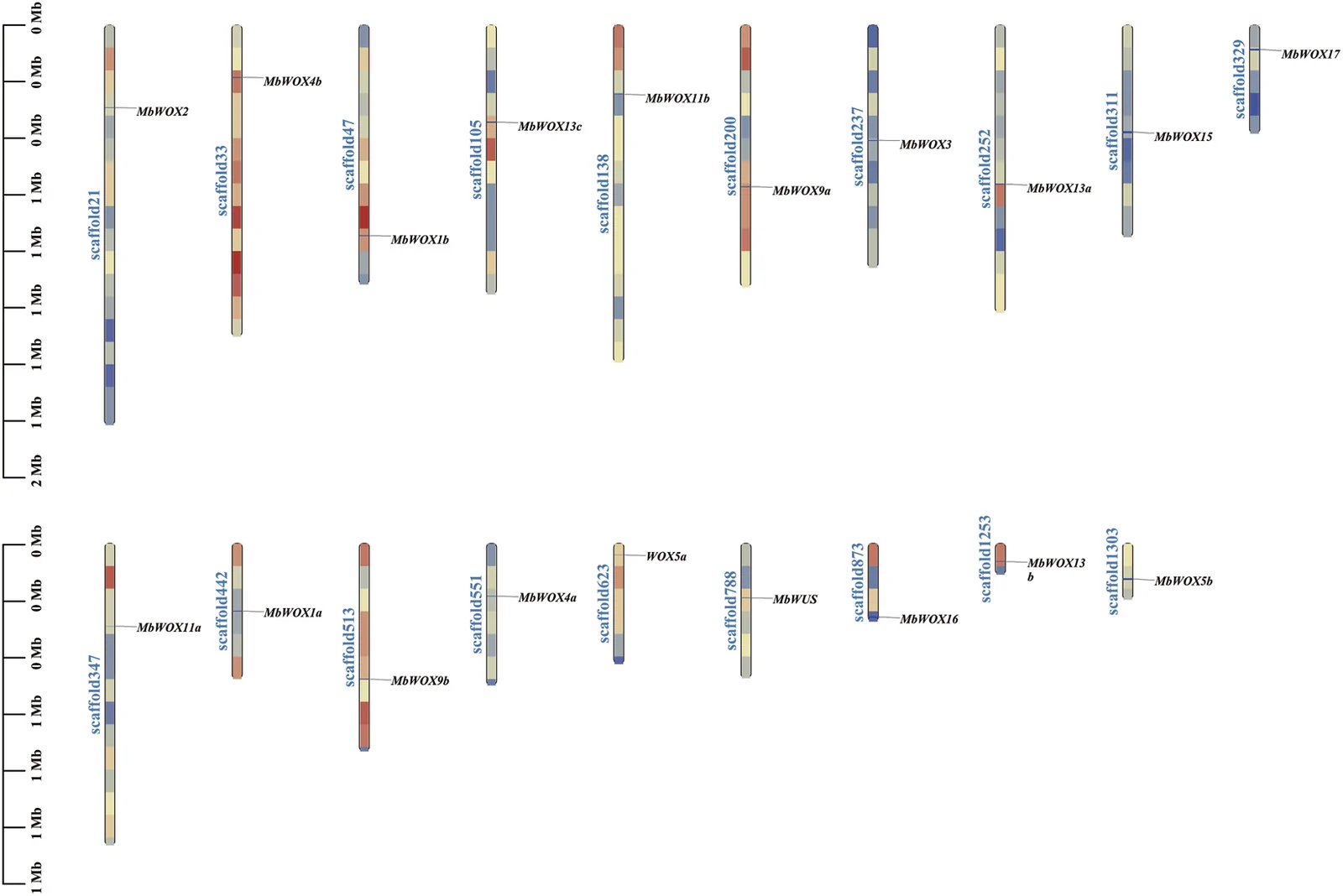
Chromosomal localization of MbWOX genes. Distribution of 19 MbWOX genes on 19 scaffold. Chromosome names are marked on bottom left of vertical bars. Scale on the left is scaffold length (Mb).

The chromosomal locations of the 19 MbWOX genes in *M. baccata* are shown in Figure 2. These genes were unevenly distributed across 19 scaffold. Overall, MbWOX genes were dispersed across multiple chromosomes without obvious clustering or chromosomal hotspots, suggesting a relatively balanced expansion pattern across the genome. The absence of significant gene enrichment in any particular chromosomal region implies that the expansion of the WOX gene family in *M. baccata* may not have been strongly influenced by local duplication events.

### 3.4 Collinearity analysis of MbWOX genes

**FIGURE 3:**
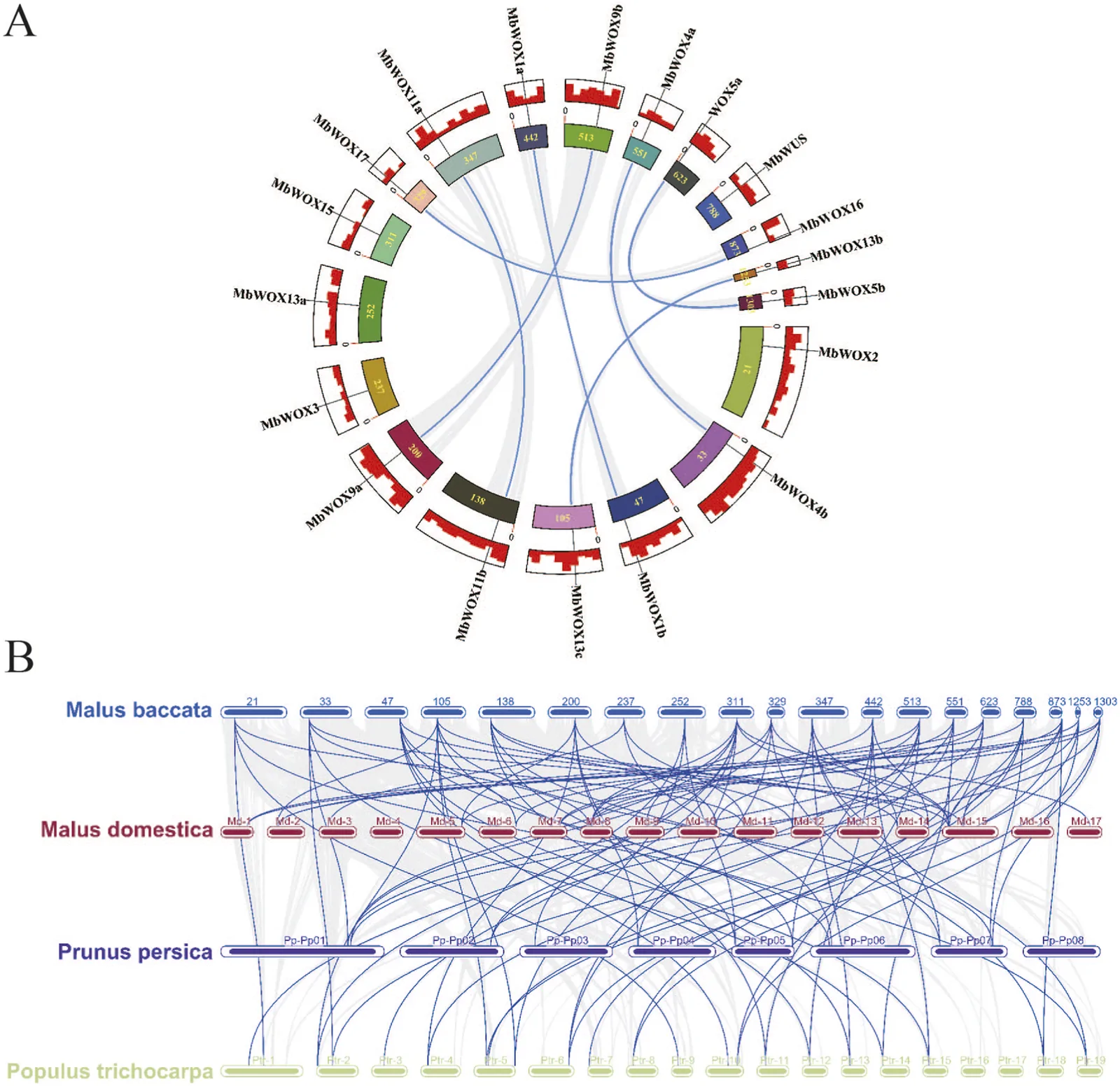
Intraspecies and interspecies synteny analysis of WOX genes in *Malus baccata*. **(A)** Intraspecies synteny analysis was conducted within the *Malus baccata* genome to identify duplicated WOX gene pairs. **(B)** Interspecies synteny analysis between *Malus baccata*, *Malus domestica*, *Prunus persica* and *Populus trichocarpa* was performed to explore the evolutionary relationships of WOX genes across species. Gray lines in the background in A and B diagrams represent all syntenic gene pairs across the genome. Blue lines in A and B diagrams represent syntenic gene pairs.

To further investigate the evolution and expansion mechanisms of the MbWOX gene family in *M. baccata*, we performed an intraspecies collinearity analysis using TBtools. The results showed that many MbWOX gene pairs had strong collinearity and were spread across different chromosomes (Figure 3A), suggesting that large-scale duplication events, such as segmental duplications or whole-genome duplications, played a major role in the expansion of this gene family. No evidence of tandem duplication was found, indicating that segmental duplication was the main driver of WOXgene expansion in *M. baccata*. To explore the conservation and evolutionary history of *WOX* genes across species, we also analyzed collinearity between the chromosomes of *M. baccata* and those of *M. domestica*, *P. trichocarpa* and *P. persica* (Figure 3B). The results revealed abundant dense collinear links between the chromosomes of *Malus baccata* and *Malus domestica*, with extensive highly conserved homologous segments across their genomes. The number and length of collinear blocks were markedly greater than those observed in intergeneric and interfamily comparison groups. These findings indicated an extremely close phylogenetic relationship between *M. baccata* and cultivated apple. No drastic remodeling of macroscopic chromosomal structures occurred during domestication, and the two species share an intact ancestral genomic framework. Abundant collinear correspondences were also detected between the two *Malus* species and peach (*Prunus persica*), demonstrating that apples and peaches diverged from a common Rosaceae ancestor, with numerous ancestral gene segments retained over long evolutionary periods within Rosaceae. In contrast to the intra-Rosaceae comparisons, collinear connections were drastically reduced and segments sparser between the two Rosaceae species and *Populus trichocarpa* (Salicaceae), with only a small number of ancient conserved homologous blocks preserved. This pattern aligns with the evolutionary rule that extensive chromosomal rearrangement and gene loss over long-term evolution gradually disintegrate collinearity after ancient lineage divergence. These results suggest that the WOXgene family is highly conserved in plants.

### 3.5 Motifs, conserved domain, and gene structure analysis of the MbWOX gene family members

**FIGURE 4:**
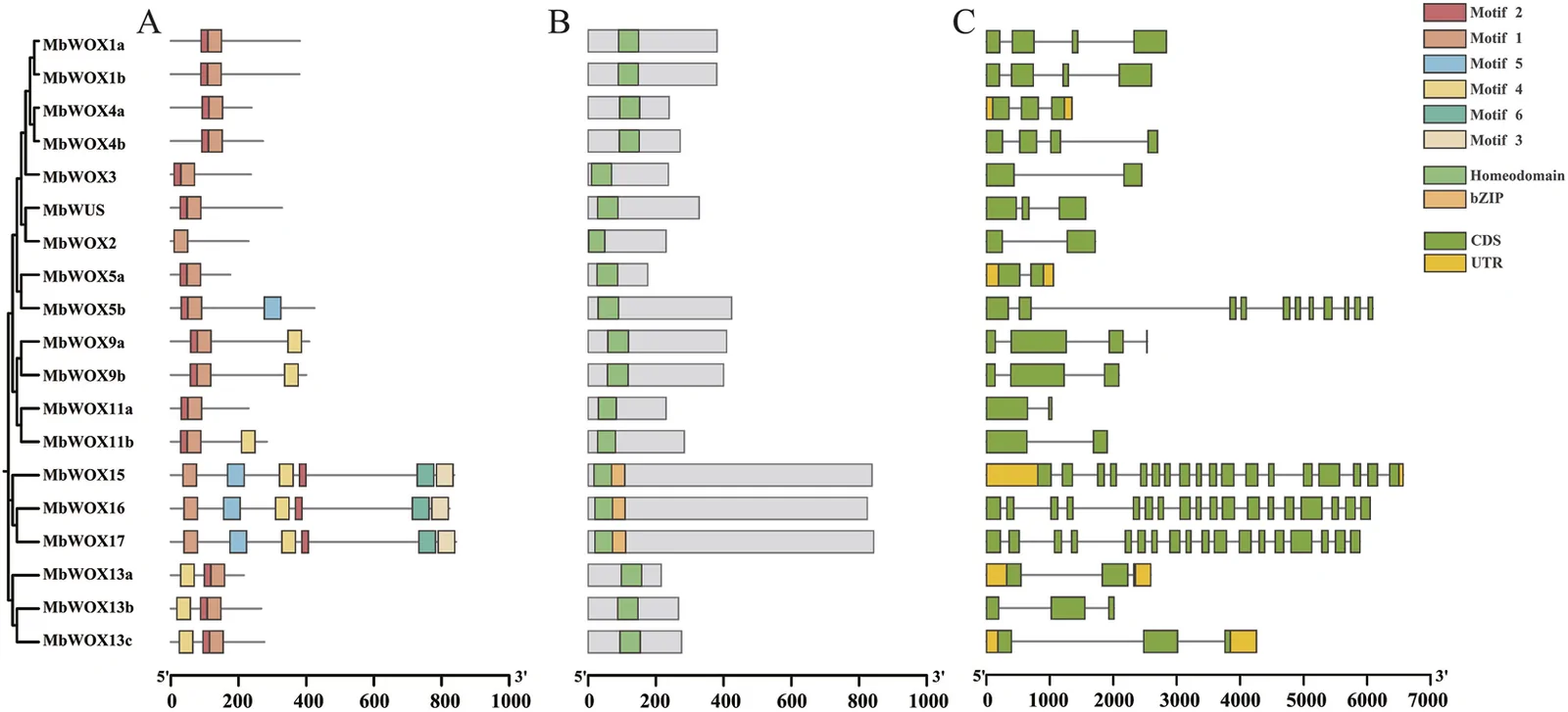
Analysis of conserved motifs, domains, and gene structures of MbWOX genes. **(A)** Colored boxes represent predicted conserved motifs. **(B)** Colored boxes represent conserved protein domains. **(C)** Colored boxes represent gene structures.

To further understand the conservation and diversity of MbWOX gene family, we analyzed their motifs, conserved domains, and gene structures. Eight motifs were identified using MEME. Among them, Homeodomain and bZIP were highly conserved across all 19 MbWOX proteins (Figure 4). Most MbWOX genes possessed both 5′UTR and 3′UTR regions, suggesting complete transcriptional regulatory information. All genes contained multiple CDS regions, and their arrangements were relatively consistent. Genes within the same clade—such as *MbWOX4a*, *MbWOX4b*, *MbWOX13a*, and *MbWOX13b*—showed almost identical gene structures and shared the same conserved domains, implying that they may have originated from gene duplication events and retained similar functions.

### 3.6 Cis-acting element analysis in the promoter regions of MbWOX genes

**FIGURE 5:**
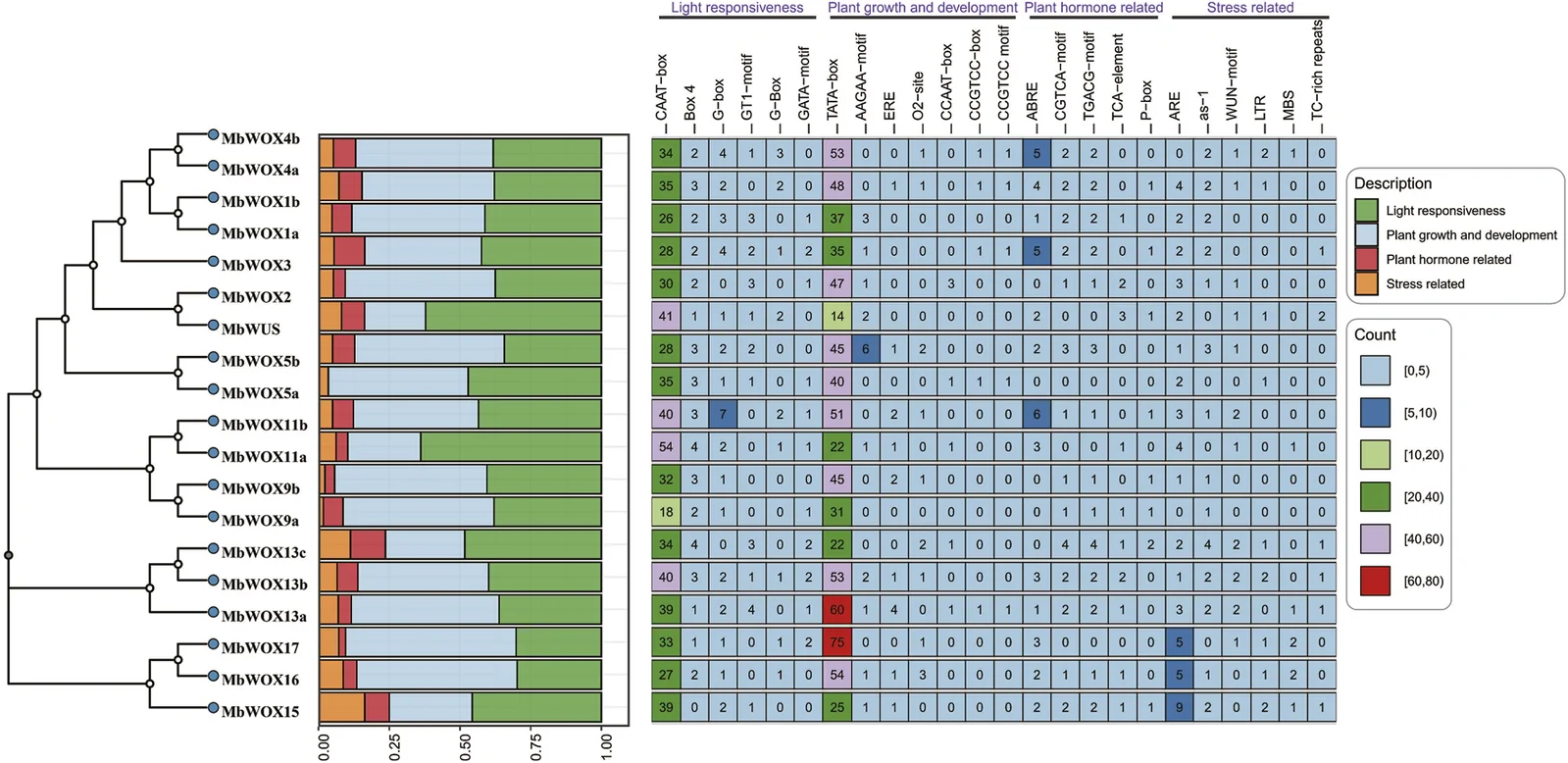
Cis-acting elements in the promoter regions of MbWOX genes. The number of each type of cis-acting element is displayed at the bottom.

In order to gain a deeper understanding of the potential regulatory mechanisms of MbWOX genes in *M. baccata*, we analyzed the 2,000 bp upstream promoter regions using the PlantCARE database. Our analysis revealed a diverse array of cis-acting elements associated with plant development, hormone signaling, and stress responses (Figure 5). These elements can be broadly categorized into four groups (1) hormone-responsive elements (e.g., ABRE, GGTCA-motif, TGA-element, CGTCA-motif): suggest that MbWOX genes may be involved in multiple hormone signaling pathways, including abscisic acid (ABA), gibberellin (GA), auxin (IAA), and jasmonic acid (JA). These pathways play vital roles in cell division, organogenesis, secondary growth, and stress responses (Thomashow, 2010; Zhou et al., 2011); (2) Stress-related elements (e.g., LTR, MBS, ARE) indicate potential roles of MbWOX genes in abiotic stress responses such as cold, drought, and hypoxia. For instance, MBS is a binding site for MYB transcription factors known to regulate drought-responsive gene (Pospíšil, 2016). (3) Light-responsive elements (e.g., G-box, GT1-motif, Box 4) are widely distributed in the MbWOX promoters, implying regulation by light signals. These elements are commonly involved in photomorphogenesis, chloroplast development, and circadian rhythm-regulated gene expression (Weyman et al., 2006; Sharma et al., 2012). (4) Plant growth and development elements such as AAGAA-motif,ERE,CCAAT-box,CCGTCC-box. In summary, the expression of MbWOX genes in *M. baccata* is likely regulated by a complex interplay of hormonal signals, environmental stresses and light conditions. The combination of these cis-elements provides a potential molecular basis for the multilayered regulation of the WOX gene family in coordinating plant growth, development, and adaptation to environmental changes.

### 3.7 Gene expression analysis of dormant buds in *M. baccata* under cold stress

**FIGURE 6:**
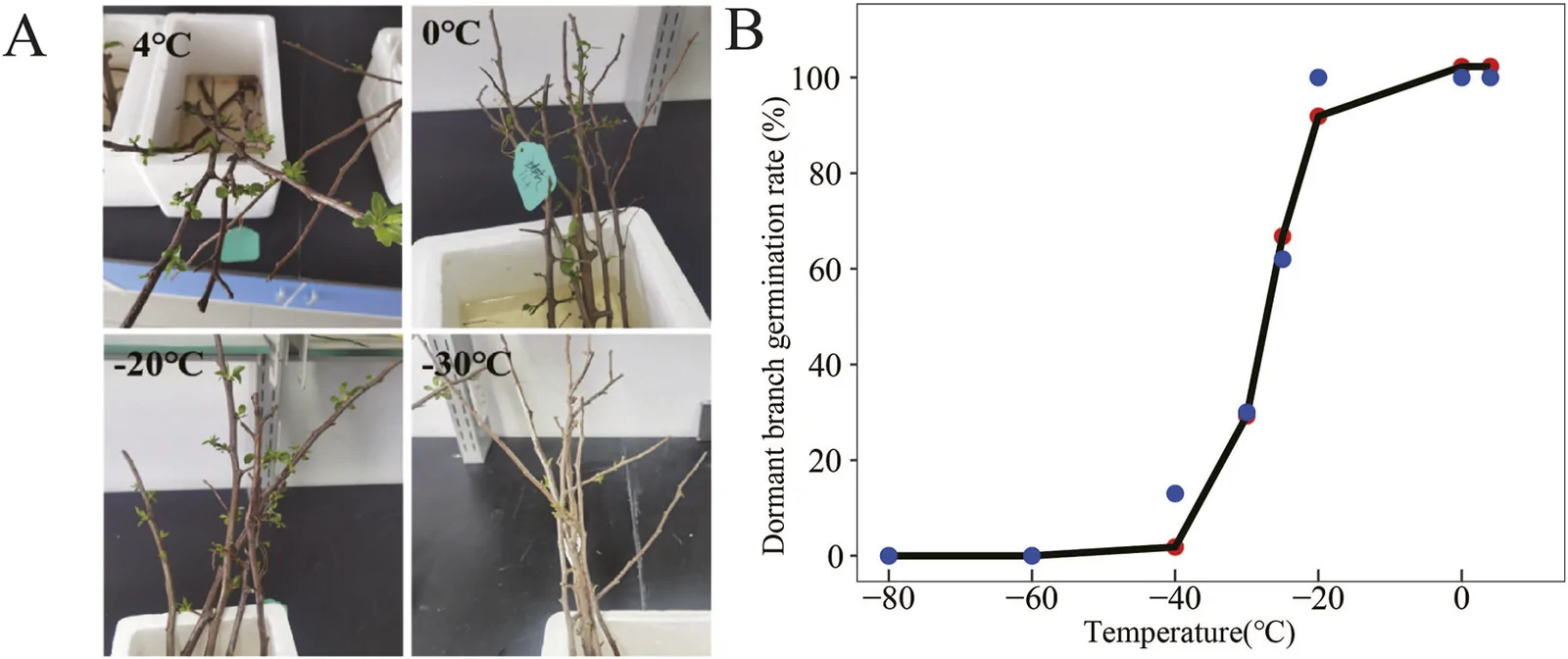
Germination rate curves of dormant shoots fitted under different temperatures. **(A)** Germination rates of dormant shoots subjected to cold treatment at varying temperatures and durations; **(B)** Germination rate curves fitted via the Logistic equation.

Dormant branches were stored at 4 °C for 5 days, followed by low-temperature treatments at 0 °C, −20 °C, and −30 °C for 7 days. Thereafter, the branches were rehydrated for bud germination rate determination (Figure 6A). A modified Logistic growth equation, established by combining the Logistic equation with an exponential equation, was used to fit the germination rate curve after low-temperature treatment (Figure 6B). The results showed that almost no bud germination occurred at approximately −40 °C.

To explore the molecular mechanism underlying gene expression patterns of dormant branches under cold stress, high-throughput RNA-sequencing was performed on dormant branches exposed to −20 °C cold stress for 0 h, 12 h, 24 h, 36 h, 48 h, 72 h and 10 days. A total of 28,981 genes were annotated. Differential expression analysis revealed that 8,598, 3,820, 8,361, 3,972, 7,296 and 7,889 differentially expressed genes (DEGs) were detected in dormant branches at 10 days, 12 h, 24 h, 36 h, 48 h and 72 h of cold stress, respectively, when compared with the 0 h control group. In addition, 5,414 DEGs were screened between the 10 days and 12 h groups. The large quantity of DEGs indicated extensive transcriptional reprogramming was triggered after 12 h of cold stress treatment.

**FIGURE 7:**
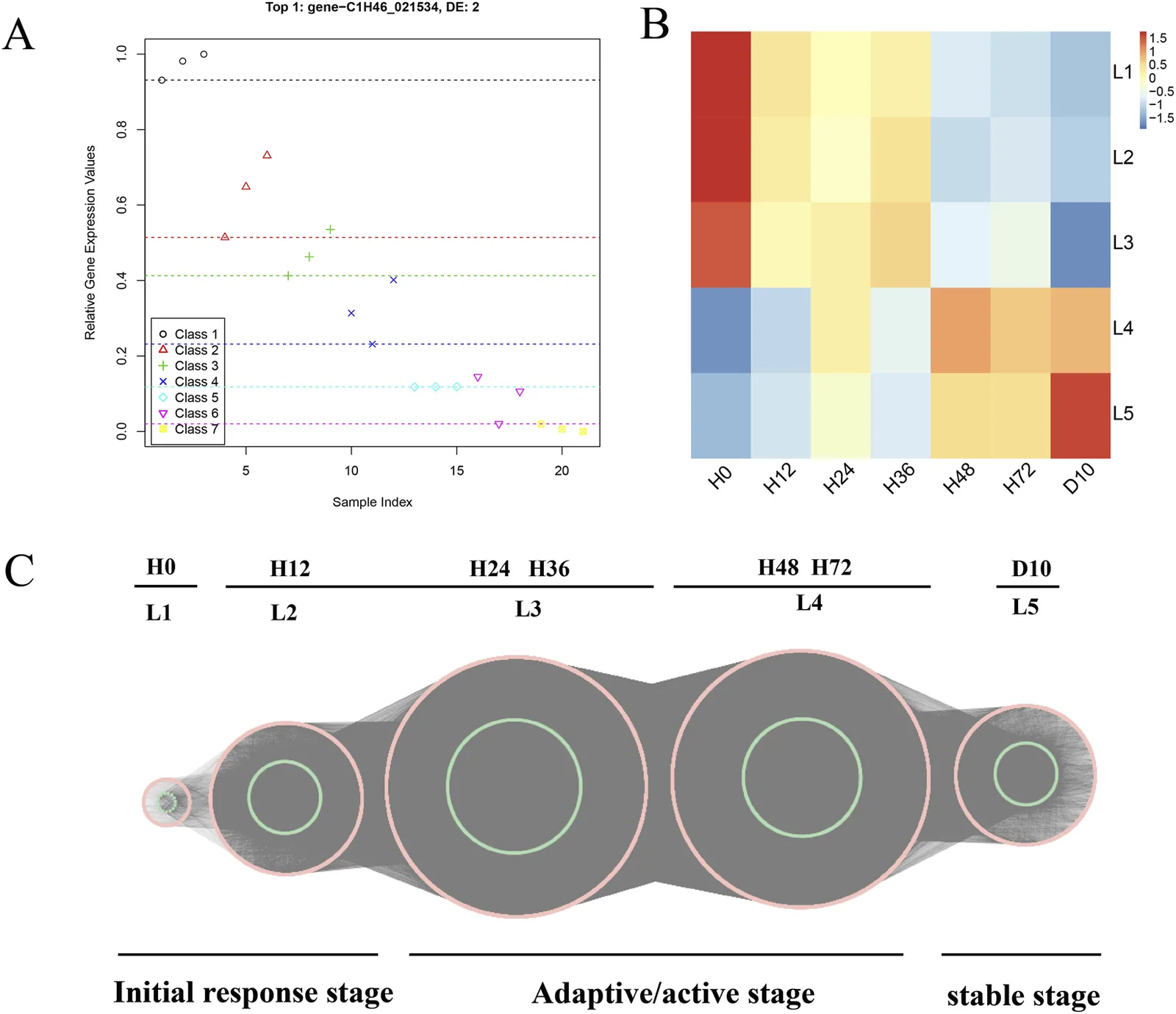
TO-GCN of transcripts from dormant shoots after cold treatment at different time points. **(A)** Relative expression levels of seed-related genes; **(B)** Heatmap of averaged normalized FPKM values for TF genes at each TO-GCN hierarchical level across all sampling time points; **(C)** TO-GCN regulatory network diagram.

**FIGURE 8:**
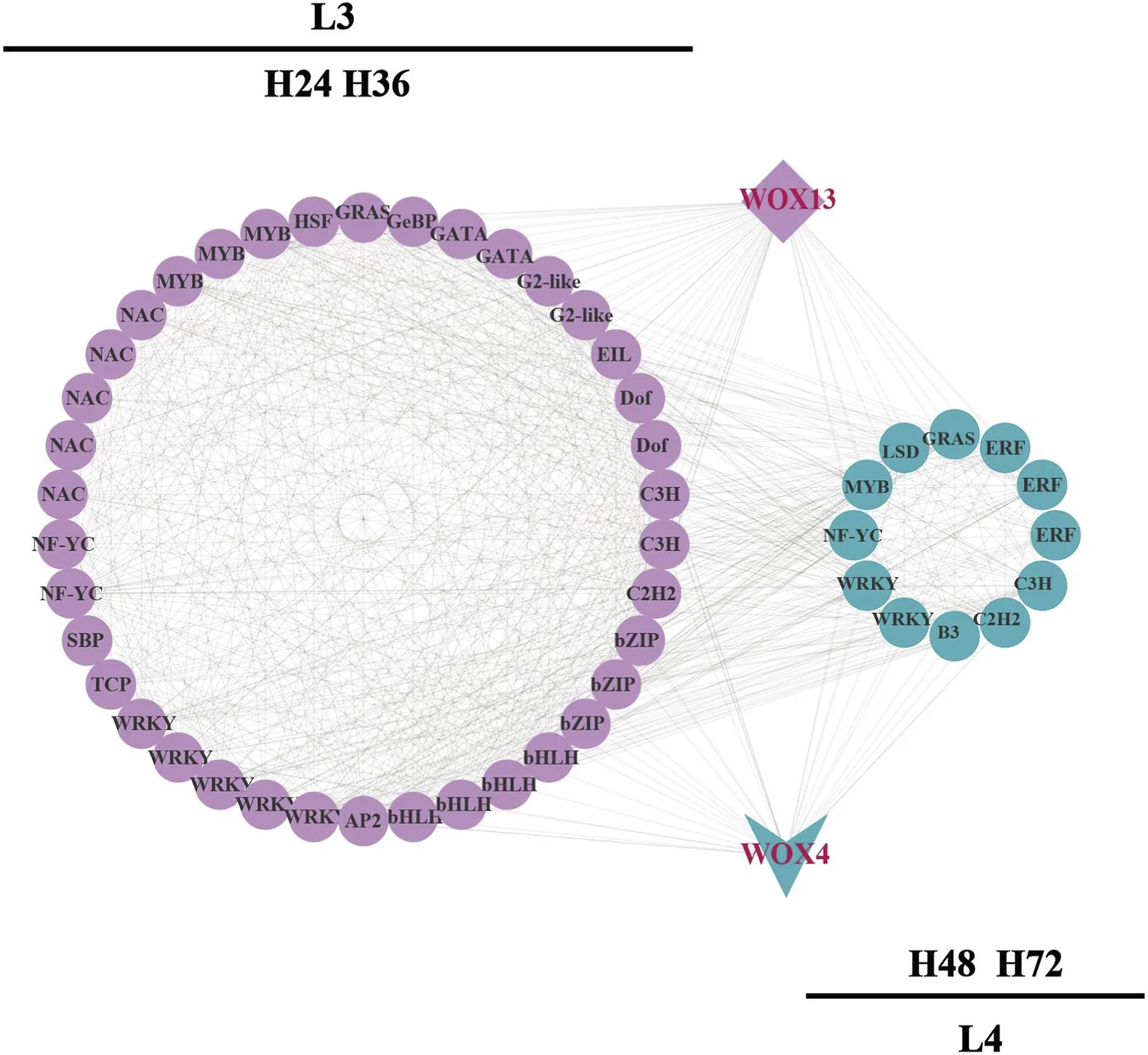
Regulatory network of WOX-related interactors. Nodes in purple represent L3 regulators, and nodes in blue denote L4 regulators.

Using gene-C1H46_021534 (annotated as *NF-YA3a*, well-documented transcription factor responsive to cold and other abiotic stresses) as the seed gene (Figure 7A), a total of 19,994 genes were used to construct the TO-GCN network with a Pearson’s Correlation Coefficient (PCC) threshold of 0.83. All genes were classified into 5 levels, 0 h corresponded to L1, 12 h to L2, 24 h and 36 h to L3, 48 h and 72 h to L4, and 10 days to L5 (Figures 7B,C). Transcription factor screening within this network demonstrated that WOX13 exhibited co-expression relationships with most transcription factor families such as MYB, NAC and bHLH, implying its involvement in the regulation of multiple signaling pathways (Figure 8). The regulatory network of WOX4 mainly interacted with specific transcription factors including ERF, GRAS and WRKY, suggesting its vital function in cold stress response (Figure 8).

**FIGURE 9:**
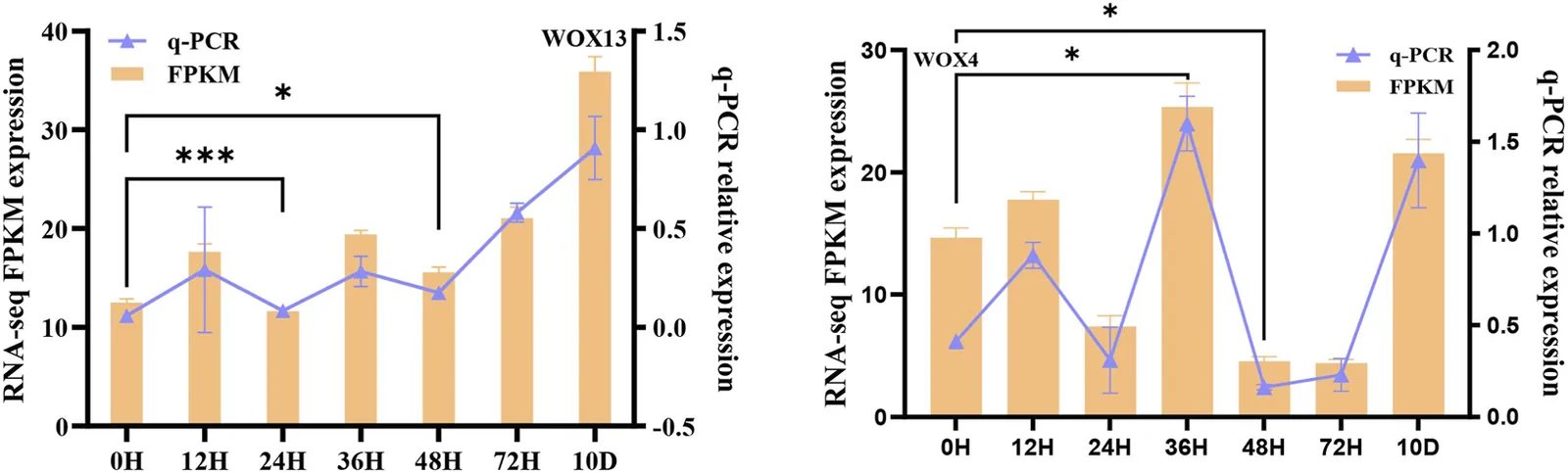
Relative expression levels of WOX13 and WOX4. q-PCR values indicate quantitative expression results, while FPKM corresponds to fragments per kilobase of transcript per million mapped reads values.

RT-qPCR was applied to verify the temporal expression patterns of *MbWOX4* and *MbWOX13* in dormant branches under low-temperature stress (Figure 9). The results showed that the expression trends of *MbWOX13* obtained by RNA-seq (Fragments Per Kilobase of transcript per Million mapped reads, FPKM) and qPCR were highly consistent, presenting an overall upward trend and reaching the expression peak at 10 days, which suggested that *MbWOX13* may play a crucial role in the late response to low-temperature stress (Figure 9A). Similarly, *MbWOX4* displayed consistent expression trends between RNA-seq and qPCR, with a typical bimodal pattern and two expression peaks at 36 h and 10 days, indicating its regulatory roles in both the early and late stages of low-temperature response (Figure 9B). The high consistency between qPCR and RNA-seq results of the two genes validated the reliability of transcriptome data. Meanwhile, it also indicated that these two WOX family genes might jointly participate in plant response to low-temperature stress via distinct temporal expression patterns.

### 3.8 Overexpression of *MbWOX13a/b* and *MbWOX4a/b* influences tolerance to low temperature stress

**FIGURE 10:**
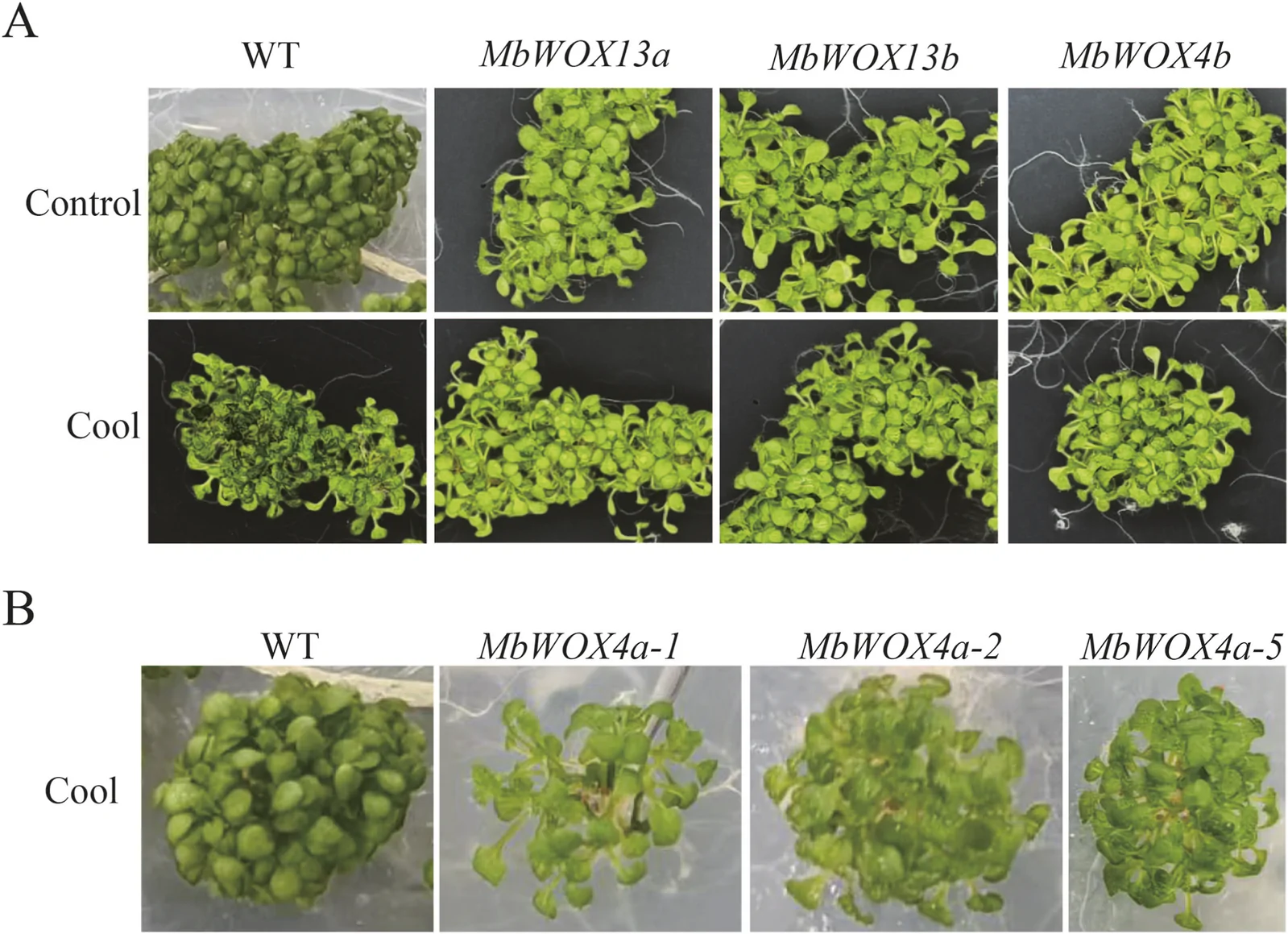
Biological functions of WOX13a/b and WOX4a/b transgenic Arabidopsis seedlings under cold stress. **(A)** Growth phenotypes of MbWOX13a/b and MbWOX4b seedlings under cold stress; **(B)** Growth phenotypes of MbWOX4a seedlings under cold stress.

To explore the functions of *MbWOX13a/b* and *MbWOX4a/b* under cold stress, F2 seeds of four overexpression lines (*MbWOX13a*-OE, *MbWOX13b*-OE, *MbWOX4a*-OE, and *MbWOX4b*-OE) were surface-sterilized and then transferred onto MS medium for cultivation. As shown in Figure 10B, two-week-old seedlings of three *MbWOX4a* overexpression lines (*MbWOX4a*-1, *MbWOX4a*-2, *MbWOX4a*-5) and the wild type (WT) were subjected to cold stress at −20 °C for 6 h. After recovery at 25 °C for 4 h, phenotypic observation and photography were performed. The results showed that the overexpression lines exhibited significantly higher degree of vitrification than wild-type seedlings. Additionally, two-week-old seedlings of *MbWOX4b*, *MbWOX13a*, *MbWOX13b* overexpression lines and WT were treated at 0 °C for 4 h, followed by recovery at 25 °C for 24 h; this treatment–recovery cycle was repeated three times. Phenotypic photography and observation revealed that the overexpression lines displayed markedly better growth performance than WT seedlings under cold stress conditions (Figure 10A).

Phenotypic observations revealed distinct differences in cold response across all transgenic lines. Lines overexpressing *MbWOX4b*, *MbWOX13a* and *MbWOX13b* displayed markedly enhanced cold tolerance and maintained superior growth after low-temperature treatment; by contrast, seedlings overexpressing *MbWOX4a* suffered more severe cold damage. The striking functional divergence between *MbWOX4a* and *MbWOX4b* indicates that these two duplicated *MbWOX4* paralogs underwent subfunctionalization during evolution, generating two isoforms with antagonistic functions in the regulatory pathway of plant cold stress response.

## 4 Discussion

In the present study, a total of 19 MbWOX genes have been identified genome-wide in cold-resistant *M. baccata*, and their physicochemical characteristics, phylogeny, chromosomal distribution, gene structure and promoter cis-elements are systematically characterized. Combined with time-series transcriptome profiling under low temperature and transgenic functional verification, *MbWOX4* and *MbWOX13* are verified as pivotal positive regulators involved in cold tolerance of *M. baccata*.

All MbWOX proteins are hydrophilic and thermally unstable with diversified molecular properties, which is consistent with previous reports on WOX family in Populus and Arabidopsis (Wang et al., 2019; Hou et al., 2021; Wang et al., 2024). The MbWOX proteins are phylogenetically clustered into three clades (ancient, intermediate, and modern), consistent with the classification established for Arabidopsis WOX family members. (Rigoulot, 2016; Cao et al., 2017), and the expansion of WUS clade is prominent in *M. baccata*, a similar evolutionary feature also observed in poplar WOX genes, implying WUS-clade expansion is closely related to environmental adaptation of woody plants. Synteny analysis demonstrated segmental duplication rather than tandem duplication dominated the expansion of MbWOX family, which is in accordance with the evolutionary pattern of MdWOX in cultivated apple (Lv et al., 2023). Strong collinear relationships among *M. baccata*, domestic apple and peach revealed high evolutionary conservation of WOX genes across Rosaceae species.

Members from the same subfamily such as *MbWOX4a/b* and *MbWOX13a/b* shared highly conserved motif composition and gene structure, originating from segmental duplication events. The conserved homeodomain existed in all MbWOX proteins, which was essential for transcription factor binding to downstream target promoters (Mayer et al., 1998). Multiple cold-, hormone- and stress-responsive cis-elements including LTR, ABRE and MBS are enriched in MbWOX promoters (Deveaux et al., 2008; Zhang et al., 2010). Wide distribution of stress-related elements further indicates MbWOX genes function in multiple abiotic stress responses.

Transgenic phenotype assays revealed divergent cold response phenotypes among these isoforms: *MbWOX4b*, *MbWOX13a* and *MbWOX13b* overexpression markedly alleviated cold damage and improved seedling survival, whereas *MbWOX4a* overexpression aggravated cold injury under low temperature. While variations in cold treatment conditions may contribute to such phenotypic differences, all assays were conducted in three independent biological replicates. Completely contrasting cold tolerance performance between *MbWOX4a* and *MbWOX4b/MbWOX13* transgenic seedlings was reproducibly detected in every replicate, which provides preliminary evidence for their opposing regulatory roles under cold stress. Previous researches about WOX-mediated cold response mostly focused on herbaceous crops, while few studies explored WOX functions in cold-tolerant *Malus baccata* (Kashyap and Forestry, 2014; Li et al., 2019). As an excellent cold-resistant germplasm surviving under −35 °C, *M. baccata* provides precious gene resources for apple cold-resistant breeding. This study clarified the cold regulatory roles of *MbWOX4* and *MbWOX13*, supplements the theoretical basis of WOX function in woody fruit trees. Further research will identify their downstream target genes via yeast one-hybrid and EMSA, and facilitate molecular breeding of cold-hardy apple rootstock.

## 5 Conclusion

In this study, we performed a comprehensive genome-wide analysis of the WOX gene family in *Malus baccata*, a crab apple with strong cold resistance widely used as apple rootstock. A total of 19 MbWOX family members were identified. Combined transcriptome profiling and RT-qPCR analysis revealed that both *MbWOX4* and *MbWOX13* were significantly induced by cold stress. The two genes displayed distinct temporal expression patterns and interacted with different transcription factor networks to mediate cold signal transduction in *M. baccata*. Transgenic functional verification further demonstrated that overexpression of *MbWOX4b*, *MbWOX13a* and *MbWOX13b* obviously improved the cold tolerance of seedlings. Notably, different isoforms of *MbWOX4* showed functional divergence under cold conditions, suggesting subfunctionalization during gene evolution.

## Data Availability

Transcriptome data were deposited to the China National Center for Bioinformation Genome Sequence Archive under accession number PRJCA067193.
